# A new approach to obtain pure and active proteins from *Lactococcus lactis* protein aggregates

**DOI:** 10.1038/s41598-018-32213-8

**Published:** 2018-09-17

**Authors:** L. Gifre-Renom, O. Cano-Garrido, F. Fàbregas, R. Roca-Pinilla, J. Seras-Franzoso, N. Ferrer-Miralles, A. Villaverde, À. Bach, M. Devant, A. Arís, E. Garcia-Fruitós

**Affiliations:** 1 Department of Ruminant Production, Institut de Recerca i Tecnologia Agroalimentàries (IRTA), 08140 Caldes de Montbui, Spain; 2 Institut de Biotecnologia i de Biomedicina, Universitat Autònoma de Barcelona, 08193 Cerdanyola del Vallès, Spain; 3 Department of Genetics and Microbiology, Universitat Autònoma de Barcelona, 08193 Cerdanyola del Vallès, Spain; 4 CIBER de Bioingeniería, Biomateriales y Nanomedicina (CIBER-BBN), 08193 Cerdanyola del Vallès, Spain; 5 Institució Catalana de Recerca i Estudis Avançats (ICREA), Barcelona, Spain; 6 Present Address: Cibbim-Nanomedicine, Hospital Vall d’Hebron, Institut de Recerca de la Vall d’Hebron (VHIR), 08035 Barcelona, Spain

## Abstract

The production of pure and soluble proteins is a complex, protein-dependent and time-consuming process, in particular for those prone-to-aggregate and/or difficult-to-purify. Although *Escherichia coli* is widely used for protein production, recombinant products must be co-purified through costly processes to remove lipopolysaccharide (LPS) and minimize adverse effects in the target organism. Interestingly, *Lactococcus lactis*, which does not contain LPS, could be a promising alternative for the production of relevant proteins. However, to date, there is no universal strategy to produce and purify any recombinant protein, being still a protein-specific process. In this context and considering that *L. lactis* is also able to form functional protein aggregates under overproduction conditions, we explored the use of these aggregates as an alternative source of soluble proteins. In this study, we developed a widely applicable and economically affordable protocol to extract functional proteins from these nanoclusters. For that, two model proteins were used: mammary serum amyloid A3 (M-SAA3) and metalloproteinase 9 (MMP-9), a difficult-to-purify and a prone-to-aggregate protein, respectively. The results show that it is possible to obtain highly pure, soluble, LPS-free and active recombinant proteins from *L. lactis* aggregates through a cost-effective and simple protocol with special relevance for difficult-to-purify or highly aggregated proteins.

## Introduction

Recombinant proteins represent a growing market and their applications are numerous for human medicine^1^ and animal health and production^2^. To date, more than 400 recombinant proteins have been approved for human medicine and this number is expected to rise in the coming years^1,3^. Nowadays recombinant products can be based on naturally occurring biomolecules, but they can also be *de novo* designed proteins or even modular proteins with improved properties. However, proteins of interest are labile macromolecules and, in many cases, they are also prone-to-aggregate. In this context, the process to obtain recombinant soluble proteins is, in many cases, complex and time-consuming. Besides, nowadays there is no well-established universal protocol for the successful production and purification of recombinant proteins. All this has lead the research community to develop specific and in many cases cumbersome, protein-dependent production and purification strategies to reach the desired product. *Escherichia coli* has been, by far, the most widely used bacterium for recombinant protein production purposes. However, although the wide catalogue of available tools for protein production in this expression system, the presence of lipopolysaccharide (LPS) in its outer membrane limits the *in vivo* applicability of the recombinant product obtained from this Gram-negative bacterium. The presence of LPS in the recombinant product can trigger non-desired inflammatory responses once the protein has been administered, making the addition of extra purification steps essential to completely remove LPS from the recombinant product^4–6^.

In the last decades, the use of alternative bacterial expression systems lacking endotoxins has significantly increased^7^. Among them, the Gram-positive *Lactococcus lactis* has been vastly studied^7–10^. This bacterium has been classified as a generally recognized as safe (GRAS) microorganism by the Food and Drug Administration (FDA) and thus, it represents a promising alternative to *E. coli* for recombinant protein production purposes^10^. Thus far, *L. lactis* has essentially been explored as a microbial cell factory for the production of soluble proteins, either intracellularly or secreted to the media^7,8,11–17^. However, recent studies show that this GRAS expression system does not differ from others in its capacity to form protein aggregates under overexpression conditions^9,18^. Different heterologous proteins produced in *L. lactis* have been found not only in their soluble form, but also as protein aggregates in the bacterial cytoplasm^19^. *L. lactis* aggregates (or inclusion bodies -IBs-), as it occurs with those found in other recombinant expression systems, are fully functional protein nanoclusters that are spontaneously formed under overproduction conditions^9,18,19^. The formation of such protein deposits is particularly relevant for those proteins difficult-to-purify and/or prone-to-aggregate^9,18^. In these cases, although the recombinant protein is free of LPS, the strategy used to produce and purify each specific protein is still largely protein-specific, as it occurs in other expression systems. Since these aggregates might be an alternative source of difficult-to-obtain proteins in *L. lactis* and considering the need to develop a universal protocol for the production and purification of LPS-free recombinant proteins, the objective of this study was to develop a broad-application strategy to extract functional protein from *L. lactis* aggregates. For that, two proteins have been used as model proteins in this work: mammary serum amyloid A3 (M-SAA3), a difficult-to-express protein^20,21^ and metalloproteinase 9 (MMP-9), which is prone-to-aggregate^18^. M-SAA3 is an acute phase protein that participates in the innate immune response of the mammary gland. On the other hand, MMP-9 is an enzyme that degrades the extracellular matrix and is involved in the immune response and tissue remodeling.

## Results

### Characterization of the M-SAA3 production in *L. lactis*

**Figure 1:**
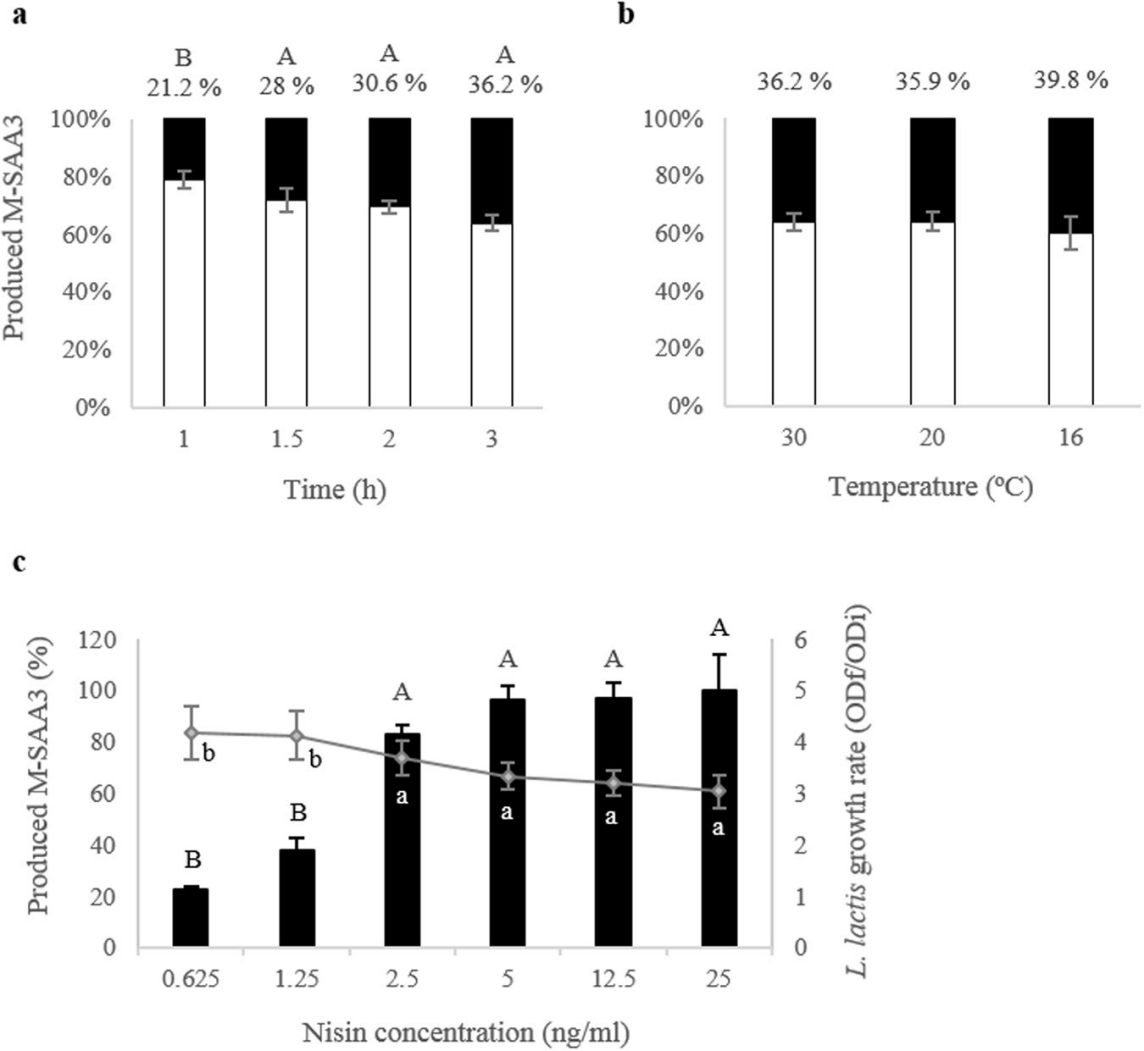
Productions of M-SAA3 in *L. lactis* after nisin induction under different conditions. (**a**) Kinetics for the produced M-SAA3 at 1, 1.5, 2 and 3 h post-induction at 30 °C. Different letters depict differences between production times (*P* = 0.002). The bars indicate the percentage of soluble (white) and of aggregated M-SAA3 (black). Error bars indicate the standard error (SE). Values for the % of aggregation are indicated on top of each bar. (**b**) Productions at different temperatures: 30, 20 and 16 °C. The bars indicate the percentage of soluble (white) and of aggregated M-SAA3 (black). Error bars indicate the standard error (SE). (**c**) Productions at different nisin concentrations, along 1.5 h and at 30 °C. The light grey line indicates *L. lactis* growth rates (final OD/OD at induction). The greatest production values of M-SAA3 were set as 100%. Different letters depict differences between nisin concentrations (*P* = 0.002) and growth rates (*P* = 0.041). Error bars indicate the standard error (SE).

In a first approach to evaluate the production profile of M-SAA3 in *L. lactis*, the production kinetics of this model protein was analyzed at 30 °C at different times post-induction. The separation of the soluble and the insoluble fractions of the cell lysate indicated that M-SAA3 was mainly produced in the soluble form (65–80%) in *L. lactis* cytoplasm, although protein aggregates (IBs) were also formed (Fig. 1a). Along time, there was a significant increase in the percentage of the aggregated M-SAA3 at 1.5, 2 and 3 h compared with 1 h post-induction (*P* = 0.002) (Fig. 1a). However, the percentage of aggregation did not change when using different growing temperatures (∼36% at 30 °C and 20 °C and 39.8% at 16 °C) (Fig. 1b).

The total amount of M-SAA3 produced improved by increasing the inducer concentration. Specifically, significant differences were observed using concentrations of 2.5, 5, 12.5 and 25 ng/ml nisin (*P* = 0.002) (Fig. 1c). Interestingly, *L. lactis* growth rate was lower in the last four nisin concentrations tested (*P = *0.041), where the M-SAA3 production was enhanced, which indicates that growth rate can be negatively affected either by high nisin concentrations or by high production of M-SAA3 (Fig. 1c).

Thus, after this first screening, the production of soluble M-SAA3 at 30 °C at 1.5 h using 12.5 ng/ml nisin was established as the optimal condition.

### M-SAA3 purification from the soluble fraction

**Figure 2:**
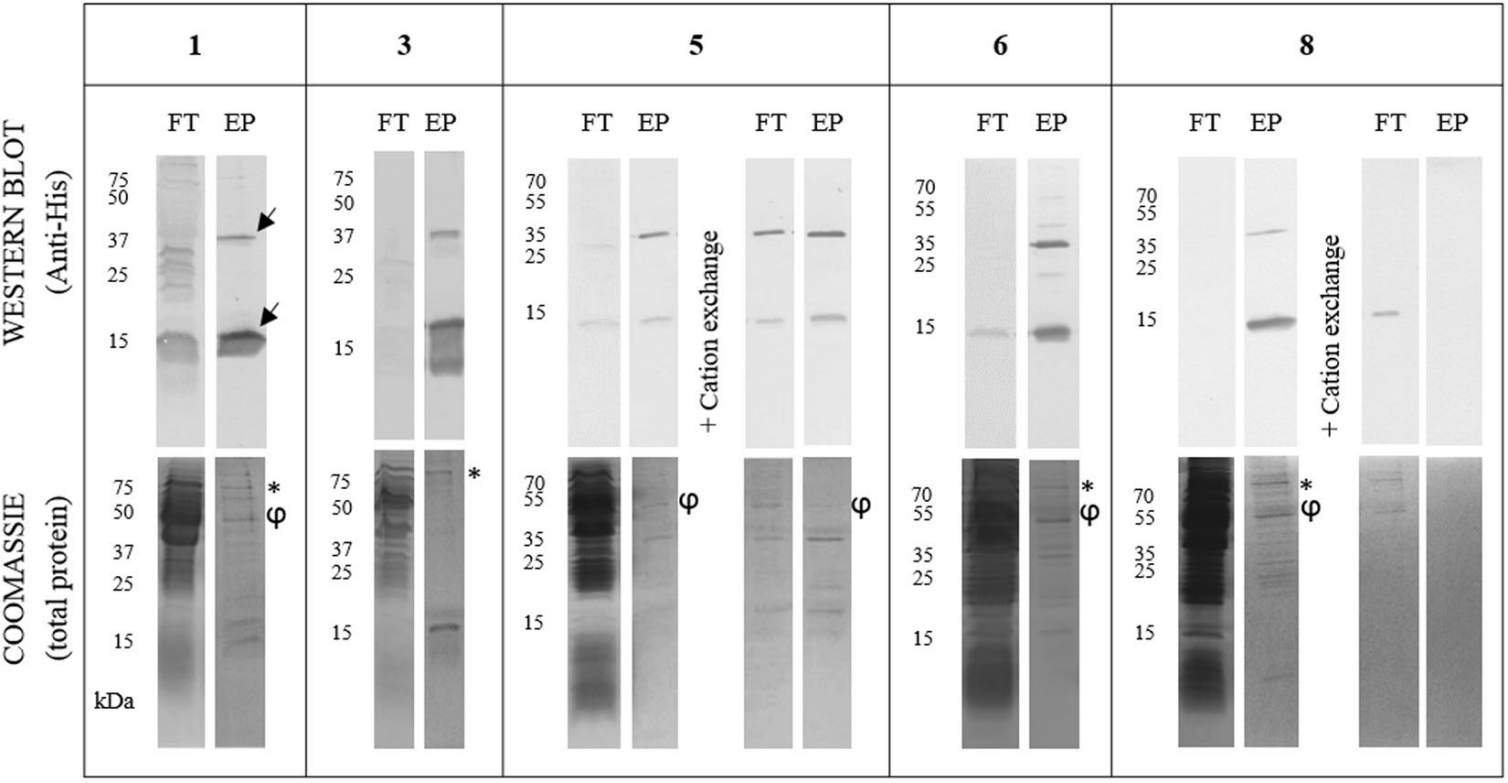
Protein gels for the soluble M-SAA3 IMAC purifications at different conditions (1, 3, 5, 6 and 8 in Table 1). Lane 1: flow through (FT) or non-bound protein, lane 2: eluted protein (EP). The eluted M-SAA3 is observed by Western blot in two bands, a monomer at 13–15 kDa and a dimer at 26–30 kDa (indicated by arrows). The obtained purities can be observed by Coomassie staining (* and φ indicate the main impurities). High-contrast was applied when necessary -only in Coomassie lanes- to allow a better display of the bands. Complete and original gels and blots for each condition can be found in the supplementary material (Supplementary Fig. 1).

As a starting point, the production and purification of soluble M-SAA3 following a standard protocol was assessed. For that, 1 l of *L. lactis* was grown at 30 °C and, after that, a purification trial using a standard immobilized metal affinity chromatography (IMAC) purification protocol for the isolation of cytoplasmic soluble proteins was performed (Table 1, condition 1). Under this condition, a large protein loss was observed during the purification process (Fig. 2, condition 1). Moreover, the eluted protein had low purity, being the presence of two proteins of around 50 and 75 kDa especially relevant (Fig. 2, condition 1). MALDI-TOF analyses revealed that these two main impurities were proteins from host bacteria and corresponded to *L. lactis* elongation factor TU (pI = 4.89; 43.2 kDa) and *L. lactis* elongation factor G (pI = 4.75; 77.9 kDa).

**Table 1:** Tested conditions in the process to optimize the purification of the soluble M-SAA3.

	1	2	3	4	5	6	7	8
Binding buffer (BB)	20 mM Tris pH = 8, 500 mM NaCl, 20 mM IMZ						20 mM Phosp. pH = 8, 1.5 M NaCl, 50 mM IMZ	20 mM Phosp. pH = 8, 500 mM NaCl, 20 mM IMZ
Elution buffer (EB)	20 mM Tris pH = 8, 500 mM NaCl, 500 mM IMZ	20 mM Tris pH = 8, 500 mM NaCl, 2 M IMZ	20 mM Tris pH = 8, 500 mM NaCl, 1 M IMZ				20 mM Phosp. pH = 8, 1.5 M NaCl, 1 M IMZ	20 mM Phosp. pH = 8, 500 mM NaCl, 1 M IMZ
Solubility Enhancer	.	.	0.5% Triton X-100 (toxic)	5% Glycerol		10% Glycerol		0.5% Tween-20
Protein loss	+++	+++	++	+++	+++	++	++	−
Cation Exchange	.	.	.	.	BB: 20 mM Tris pH = 7 EB: 20 mM Tris pH = 7, 1 M NaCl	.	.	BB: 20 mM Tris pH = 8 EB: 20 mM Tris pH = 8, 1 M NaCl
Purity (%)	13.8	12.4	54	15.2	30.4	20.8	ND	ND
Main Impurities	50–75 kDa	50–75 kDa	75 kDa	50 kDa	50 kDa	50–75 kDa	50–75 kDa	50–75 kDa

IMZ: imidazole; Phosp.: phosphate buffer; pI: Isoelectric point; ND: non-detectable.

Aiming to optimize the purification process of the soluble protein M-SAA3, eight different strategies combining different imidazole concentrations and/or the addition of detergents or other solubility enhancers were tested (Table 1, conditions 2–8**)**. In some cases, a cation-exchange step was added after the IMAC purification (Table 1, conditions 5 and 8) in order to increase the purity of the recombinant product. Some strategies such as conditions 3, 6, 7 and 8 reduced the protein loss, being this improvement particularly relevant for the condition 8. However, despite a substantial decrease in the amount of unbound protein, none of these strategies (conditions 3, 6, 7 and 8) significantly increased the purity of the final product. Besides, the incorporation of a purification step using a cation-exchange approach did not improve the result of the whole purification process. Thus, in general terms we can conclude that none of the tested strategies allowed to obtain highly pure M-SAA3 through an efficient purification process (Fig. 2).

### Solubilization and purification of M-SAA3 aggregates

**Figure 3:**
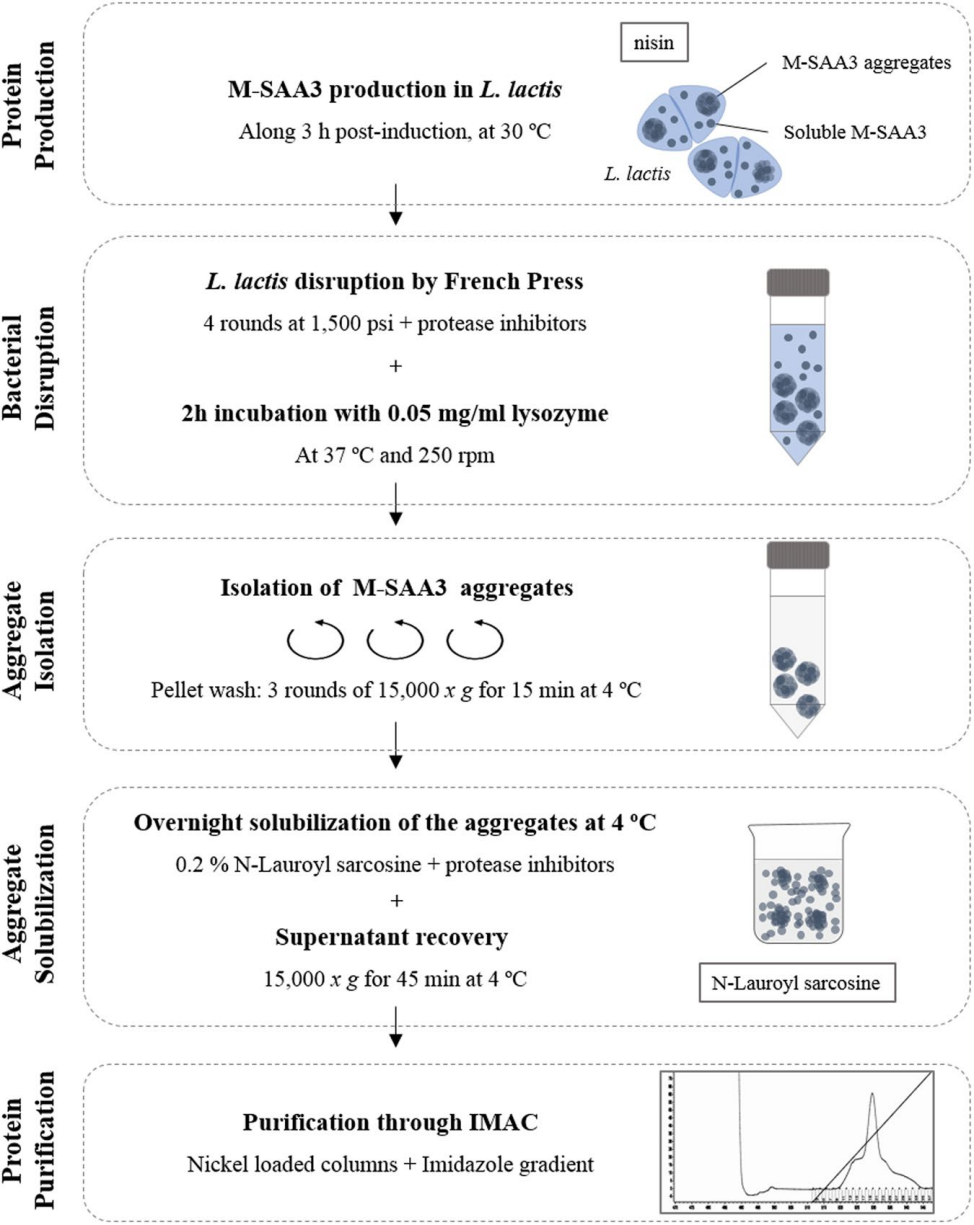
Schematic protocol for the solubilization of the M-SAA3 IBs produced in *L lactis*.

**Figure 4:**
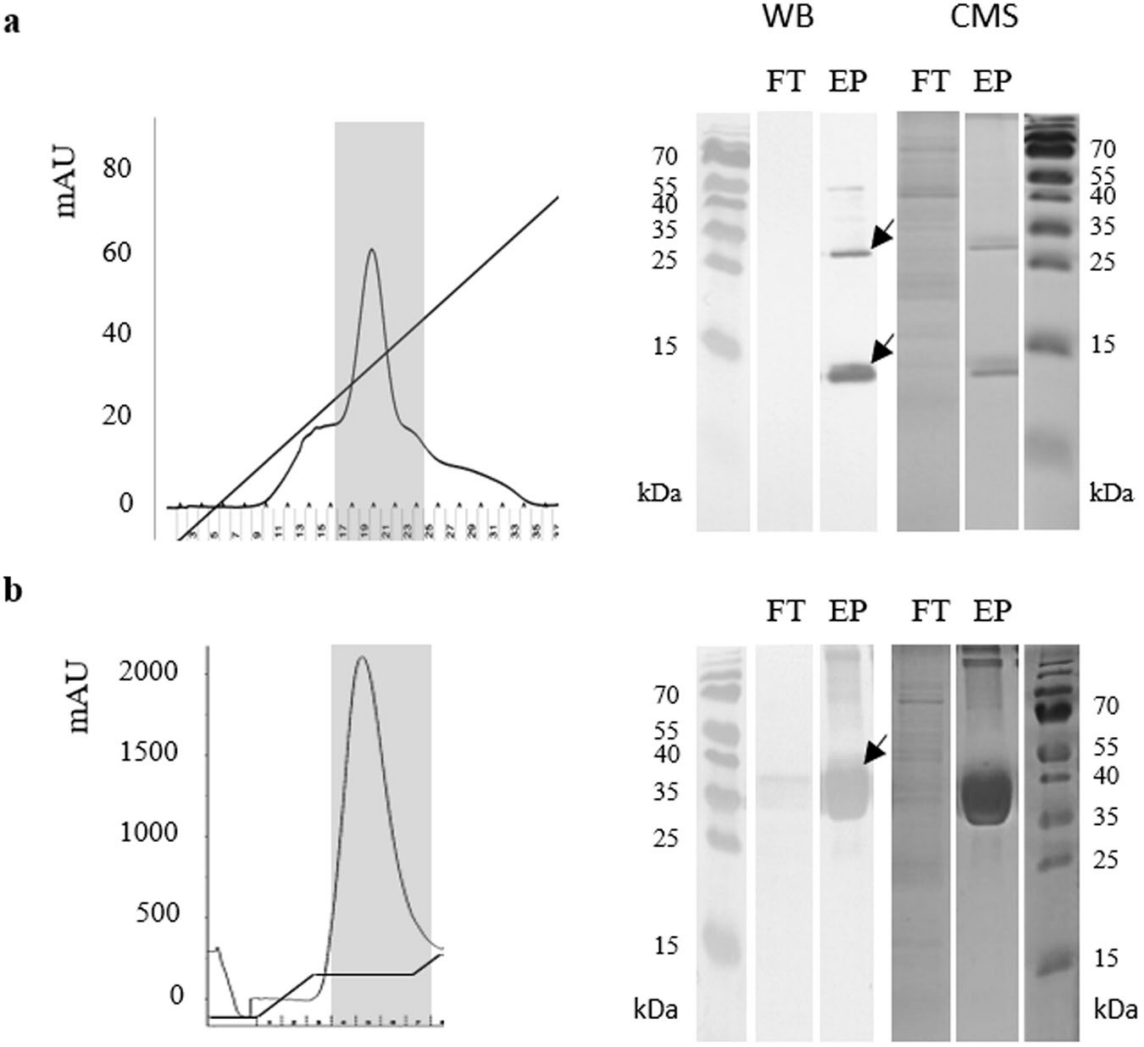
IMAC purification of the solubilized aggregates produced in *L. lactis*. In the left side, the chromatograms of the M-SAA3 (**a**) and MMP-9 (**b**) elutions. In grey, the selected fractions of the eluted proteins. In the right side, Western Blot and Coomassie staining for each eluted protein (EP) and flow through (FT). The arrows indicate the correspondent bands for each protein -the M-SAA3 (a) appears in a monomeric and a dimeric form, whereas MMP-9 (b) appears mainly as monomers-. Complete gels and blots can be found in the supplementary material (Supplementary Fig. 2).

Once proven that soluble M-SAA3 produced in *L. lactis* cannot be successfully purified using conventional strategies under a trial-and-error process, we evaluated whether the extraction of soluble M-SAA3 from *L. lactis* aggregates could be an alternative. Considering that *L. lactis* aggregates are formed by biologically active proteins, a new, simple and non-denaturing protocol for the isolation of soluble and functional proteins using these bacterial aggregates as protein source was developed. Specifically, different washing steps with the use of a mild detergent for the efficient solubilization of M-SAA3 IBs, without using any denaturing agent, were combined (Fig. 3). Importantly, we proved that is possible not only to isolate soluble M-SAA3 protein from *L. lactis* aggregates, but also that the purified product has a high degree of purity (>98.5%) (Fig. 4a). In this context, it is important to emphasize that the recombinant product was purified in absence of the two major impurities (*L. lactis* elongation factor TU and elongation factor G) found in most of the conditions tested for the purification of soluble protein (Table 1). In the elution profile of the purification of the solubilized M-SAA3, only two bands of 13–15 kDa and 26–30 kDa were detected, which corresponded to M-SAA3 monomer and dimer, respectively (Fig. 4a).

### Activity of the solubilized and purified M-SAA3

**Figure 5:**
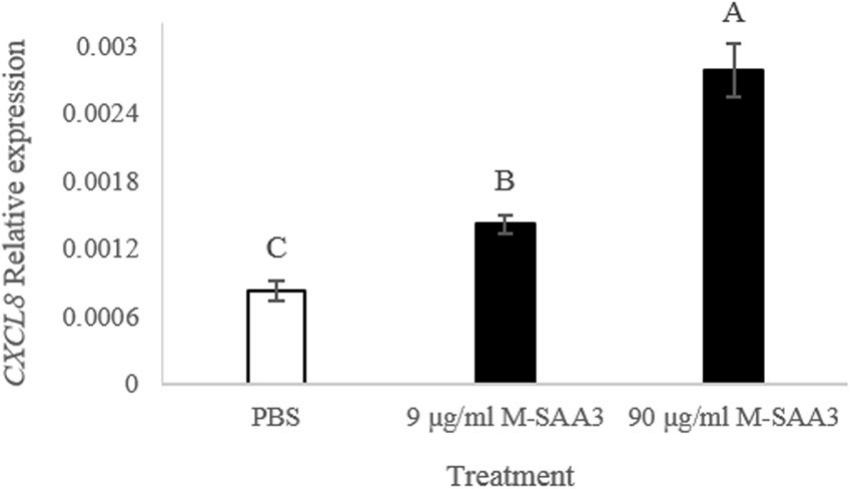
*CXCL8* gene expression by bovine epithelial cells from the mammary gland with solubilized and purified M-SAA3, obtained from *L. lactis* aggregates*. CXCL8* gene expression using 9 µg/ml and 90 µg/ml dose, using PBS as a control. Different letters depict differences between treatments (*P* < 0.0001). Error bars indicate the standard error (SE).

An *in vitro* assay using bovine epithelial cells from the mammary gland was conducted to test the activity of the M-SAA3 obtained through the new purification method. The results of this assay showed that the M-SAA3 was fully active and a dose-dependent effect could be observed on the stimulation of interleukin 8 (*CXCL8*) expression (Fig. 5) after M-SAA3 treatment^21^. Specifically, a 1.6-fold increase of *CXCL8* expression compared with the PBS treatment was obtained by adding 9 µg/ml of M-SAA3 to the cells, whereas a 3-fold increase was noted for the treatment with 90 µg/ml of M-SAA3 (*P* < 0.0001).

### IB solubilization of prone-to-aggregate proteins

**Figure 6:**
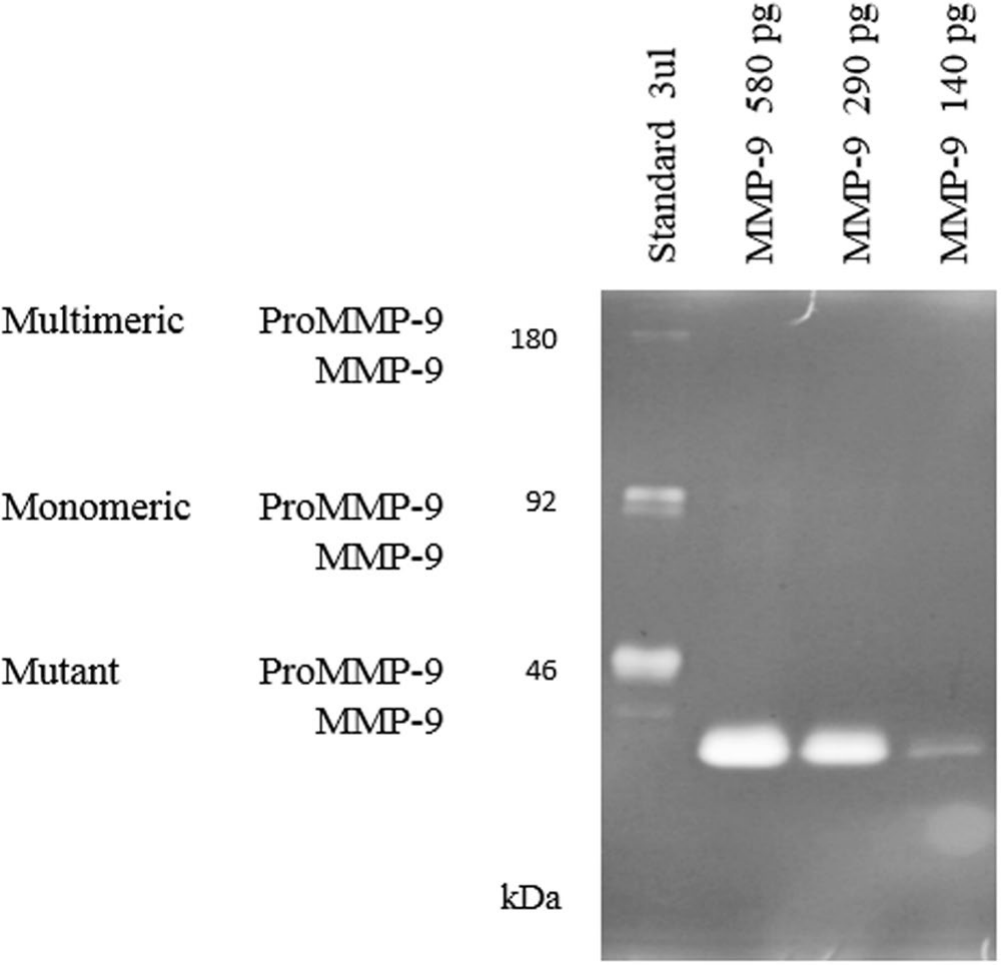
Zymography of MMP-9. Gelatin degradation can be observed using three different amounts of the solubilized MMP-9. Line 1: standard; line 2–4: different amounts of soluble MMP-9. The molecular weight of the solubilized MMP-9 (catalytic domain) is 39 kDa.

To determine the efficacy of the new protocol with other difficult proteins, we assessed its effectiveness with a protein with a high tendency to aggregate, namely MMP-9. The aggregation rate in *L. lactis* of this protein has already been described, with reported values up to 100%^18^. Thus, we produced and purified MMP-9 aggregates, which were solubilized following the described protocol (Fig. 3). Again, soluble and highly pure proteins were obtained with this novel protocol. Specifically, a purity of 99% was observed by Coomassie staining for MMP-9 (Fig. 4b) and the activity of the soluble metalloproteinase obtained through the new method was tested. Specifically, the activity was tested by zymography, observing that soluble MMP-9 was active following a dose-dependent effect (Fig. 6).

## Discussion

The production and purification of recombinant proteins is often a difficult and product-dependent process. The M-SAA3 is just one example amongst many other difficult-to-produce and difficult-to-purify proteins. Previous reports have shown that its production requires a difficult and a time-consuming process^20–22^. Moreover, results shown in this work reveal that none of the commonly used strategies to improve protein production and purification can be used for the isolation of M-SAA3 in its soluble form, using *L. lactis* as an intracytoplasmic expression system (Fig. 2). Another example of difficult proteins are MMPs, which have previously shown to have a strong tendency to aggregate in both *E. coli*^23–25^ and *L. lactis*^18^, reaching levels of up to 100% aggregation. Under these situations, which are common under recombinant production conditions, protein production and purification from the soluble fraction can become a long and unsuccessful trial-and-error process and alternative approaches are needed.

IBs are protein aggregates produced in recombinant bacteria under conditions of protein overproduction^26,27^. Their formation has been mainly described in *E. coli* cytoplasm, but increasing evidence shows that aggregation under these conditions occurs in different expression systems, including *L. lactis*^28^. These aggregates are composed mainly by the overexpressed protein in its active form and they are formed through a specific process^29,30^, which means that they might drag just few proteins from the cell cytoplasm. Thus, due to their special features, IBs represent an appealing source of functional recombinant protein^31^. However, traditionally, the recovery of proteins from IBs produced by *E. coli* has been done using a two-step process, including a hard denaturation step (using agents like urea or guanidine hydrochloride) followed by, an often, non-efficient refolding process^32,33^. This aggressive treatment disregards the IB nature and protein comprised in these aggregates is completely denatured. Thus, since these aggregates are formed by functional recombinant protein, the use of mild protocols to extract properly folded soluble proteins from IBs could be a promising strategy to avoid the use of denaturing agents. In this context, this approach has already been proven in *E. coli*^32,34^, but no procedure has been developed thus far for the isolation of soluble and LPS-free proteins using *L. lactis* aggregates^14,18^. In the present work, we have developed a novel and straightforward procedure (Fig. 3) to obtain soluble, pure and biologically active proteins from *L. lactis* aggregates. Since the formation of MMP-9 IBs by *L. lactis* has been recently described^18^ and the aggregation rates of the M-SAA3 can be increased by prolonging the production incubation time (Fig. 1a), we have used these proteins as models to evaluate *L. lactis* aggregates as a potential source of difficult proteins. Specifically, we have proven that it can be successfully applied with both difficult-to-express and difficult-to-purify proteins (Figs 4–6). On the one hand, we have shown that protein purity levels increase (Fig. 4) and the recovered protein is biologically active (Figs 5 and 6). Moreover, unlike protocols developed for the isolation of soluble proteins from *E. coli* IBs, this new procedure allows to isolate LPS-free proteins. Altogether gives consistency to the new method that can be applied as a unique approach to obtain pure yields of difficult-to-isolate proteins from bacterial aggregates in a soluble format and free of endotoxins. Thus, this approach is an appealing alternative to the extracytoplasmatic production^35^ for the isolation of soluble proteins that are mainly produced as protein aggregates.

## Conclusions

We have developed a new approach to obtain prone-to-aggregate and difficult-to-purify proteins in its soluble form using *L. lactis* as expression system. For the first time, we have proven that it is possible to use *L. lactis* aggregates as a source of fully functional proteins free of endotoxins. For that we have developed a protocol widely applicable for proteins that cannot be obtained through standard procedures without the need to add denaturing agents. The developed method represents an economical alternative that opens the door to the production of new recombinant proteins that, to date, could not be obtained.

## Methods

### Bacterial strains and plasmids

*Lactococcus lactis subsp. cremoris* NZ9000^36^ and NZ9000 *clpP*^−^ *htrA*^−^ (*clpP-htrA*; erythromycin resistant (Em^R^))^37,38^ (kindly provided by INRA, Jouy-en-Josas, France; patent n° EP1141337B1) strains and the sequence of two proteins, the catalytic domain of bovine metalloproteinase 9 (MMP-9)^18^ and the goat mammary serum amyloid A3 (M-SAA3)^21^, were used in this study. Each protein sequence was flanked by *Nco*I and *Xba*I restriction sites and C-terminally fused to a His-tag for purification and quantification purposes. All genes were ligated into the chloramphenicol resistant (Cm^R^) pNZ8148 plasmid (MoBiTech). The plasmid pNZ8148:SAA3 was transformed into electrocompetent *L. lactis* NZ9000, while pNZ8148:MMP-9 was transformed in *L. lactis clpP-htrA*. For electroporation, a Gene Pulser (Bio-rad) at 2500 V, 200 Ω and 25 µF was used as detailed by Cano-Garrido *et al*.^18^.

### M-SAA3 protein production

*L. lactis* NZ9000/pNZ8148:SAA3 plasmid was grown overnight (O/N) at 30 °C in GM17 with 5 µg/ml Cm for plasmid maintenance. The O/N cultures were inoculated in fresh GM17 with Cm at an initial optical density at 600 nm (OD_600_) of 0.1. When cultures reached an OD_600_ = 0.4–0.6, the recombinant protein expression was induced with 12.5 ng/ml nisin.

Culture samples of 25 ml were taken and centrifuged (6,000 × *g*, 15 min, 4 °C) at 0, 1, 1.5, 2 and 3 h post-induction for a protein kinetics analysis. Samples for a temperature-effect analysis were taken at 0 and 3 h post-induction from cultures grown at 30 °C and at 0 and 16 h post-induction from cultures grown at 20 and 16 °C. The inductor concentration-effect analysis was assessed by samples taken at 0 and 3 h post-induction with seven different nisin concentrations (0.625, 1.25, 2.5, 5, 12.5 and 25 ng/ml) in cultures grown at 30 °C. All the experiments were run in triplicate.

In all cases, pellets were resuspended in 500 µl PBS with an EDTA-free protease inhibitor cocktail (Roche) and bacteria were disrupted by sonication. Each sample was ice-coated and sonicated with 2 cycles of 1.5 min (0.5 sec cycles at 10% amplitude). The soluble and the insoluble protein fractions were separated by centrifugation at 15,000 × *g* for 15 min at 4 °C and the insoluble fraction was resuspended in the same buffer at the same initial volume.

### Protein determination

The soluble and the insoluble protein fractions were analyzed by 15% denaturing sodium dodecyl sulfate polyacrylamide gel electrophoresis (SDS-PAGE). All samples were resuspended with Laemmli loading buffer (100 mM Tris base, 8% glycerol, 55 mM SDS, 4% β-mercaptoethanol, 1.6 M urea). Soluble fractions were boiled for 5 min and insoluble fractions for 40 min before electrophoresis. Protein bands were electroblotted into PVDF membranes at constant 250 mA and 100 V for 1 h, followed by a blocking step with BSA O/N at 4 °C (5% BSA in TBST buffer: 10 mM Tris, 150 mM NaCl, 0.05% Tween 20). Anti poly-histidine (GE Healthcare; mouse) was used as the primary antibody at a 1/1,000 dilution in BSA-TBST buffer, in which membranes were incubated along 2 h at room temperature (RT), followed by 3 washes in TBST buffer. Then membranes were incubated in a 1/20,000 dilution in TBST of an anti-mouse IgG-alkaline phosphatase (Sigma), used as secondary antibody, along 1 h at RT followed by 3 washes in TBST buffer. Protein bands were developed after adding the alkaline phosphatase substrate solution NBT/BCIP (Thermo Scientific). Bands were quantified with a standard curve of T22-GFP-H6^39^ and densitometry analyses with ImageJ software. The M-SAA3 aggregation rate (insoluble protein/total protein) in *L. lactis* was calculated for both the kinetic and the temperature experiments.

### Production and purification of the M-SAA3 from the soluble fraction

Cultures of 1 l of the M-SAA3-producer *L. lactis* were grown at 30 °C and induced with nisin at an OD_600_ = 0.4–0.6, as previously described^14^. The whole volume was recovered after 1.5 h of production and centrifuged at 5,000 *x g* for 15 min at 4 °C. Pellets from 500 ml of culture were suspended in 30 ml of the binding buffer (20 mM Tris pH = 8, 500 mM NaCl, 20 mM imidazole) with an EDTA-free protease inhibitor cocktail (Roche). Bacteria were mechanically disrupted by French Press (Thermo FA-078A) with 4 cycles at 1,500 psi in ice coating. Cell lysates were centrifuged at 15,000 × *g* for 45 min and the M-SAA3 in the soluble fraction was purified by Immobilized Metal Affinity Chromatography (IMAC) in an ÄKTA purifier FPLC (GE Healthcare) using 5 ml HiTrap Chelating HP columns (GE Healthcare).

### Optimization of the purification process of the soluble M-SAA3

Eight different purification experiments were run to optimize the M-SAA3 final purity. Different imidazole concentrations (500 mM, 1 M and 2 M) were used in the elution buffer and none or different solubility enhancers were added to both the binding and the elution buffers (Triton X-100, glycerol, or Tween 20). Also, changes in the binding buffer were conducted: an increase of the imidazole concentration from 20 to 50 mM, a replacement of the Tris buffer for a phosphate buffer and an increase of the NaCl concentration from 500 mM to 1.5 M. All the tested combinations are detailed in Table 1.

The eluted peaks were dialyzed in PBS O/N, at 4 °C and with gentle agitation, unless a cation-exchange purification was scheduled. Cation-exchange chromatography was conducted in experiments 5 and 8 (see Table 1). The peaks obtained by the IMAC purification were dialyzed in 20 mM Tris pH = 7 in experiment 5, or in 20 mM phosphate buffer pH = 8 in experiment 8 (M-SAA3 isoelectric point is 8.67). In both cases, the dialyzed peaks were loaded in a negatively charged 1 ml SP FF column (GE Healthcare) and M-SAA3 was eluted by an increasing linear NaCl gradient to 1 M final concentration. Eluted samples were dialyzed in PBS as previously described.

The recovering efficiency and the purity of the eluted samples were determined by Western blot, as previously described^14^ and by Coomassie staining.

### Solubilization of M-SAA3 and MMP-9 from protein aggregates

Five liters of M-SAA3 were produced in *L. lactis* NZ9000 and 2 l of MMP-9 were produced in *L. lactis clpP-htrA*. In these cases, inductions were conducted for 3 h. The whole volumes were centrifuged at 6,000 x *g* and the pellets were resuspended in lysis buffer (20 mM Tris, 500 mM NaCl, 20 mM imidazole, 10% glycerol) in presence of protease inhibitors and in a ratio of 500:30 (ml:ml, culture:buffer). Samples were subjected to 4 rounds of French Press disruption at 1,500 psi, intercalated by a minimum of 5 min repose in ice. After that, 0.05 mg/ml lysozyme was added and samples were incubated for 2 h at 250 rpm and 37 °C. Protein pellets were recovered and washed twice with distilled water. Pellets were weighted and solubilized in 0.2% N-lauroyl sarcosine in Tris solution at a ratio 1:40 (g:ml) as described by Peternel *et al*.^34^ and adding protease inhibitors. The mixture was incubated O/N at 4 °C in agitation and the supernatant was recovered through centrifugation at 15,000 × *g* for 45 min at 4 °C for further purification.

### Purification of the solubilized M-SAA3 and MMP-9

NaCl and imidazole were added to the solubilized proteins to equilibrate the samples with the binding buffer composition and IMAC purification was carried as previously described. Both the binding and the elution buffer contained 0.2% N-lauroyl sarcosine and the final imidazole concentration in the elution buffer was 1 M for the M-SAA3 and 500 mM for the MMP-9. The selected fractions were dialyzed in PBS O/N at 4 °C and with gentle agitation. The amount of purified protein was determined by Bradford’s assay^40^ and the integrity of the protein analyzed by SDS-PAGE.

### Identification of the main contaminating proteins

The main contaminating bands were cut and sequenced by MALDI-TOF in the Servei de Proteomica i Biologia Estructural (sePBioEs, Autonomous University of Barcelona).

### Activity of the solubilized M-SAA3

Mammary epithelial cells from primary cultures were obtained as described elsewhere^21^ and seeded in 24-well plates at 44,000 cells/well. After 24 h incubation at 37 °C and 5% CO_2_, wells were washed twice with warm PBS and 500 μl PBS or two doses (9 µg and 90 µg in 500 μl of PBS) of the solubilized and purified M-SAA3 were added to each well containing 500 μl of DMEM/F‐12 medium with 8 μg/ml bovine insulin and 50 μg/ml hydrocortisone by sextuplicate. After 3 h of incubation at 37 °C, 5% CO_2_, cells were gently washed with PBS and 500 μl of Trizol reagent (Thermo Fisher Scientific) were added to each well to collect and lysate the cells. The extraction of RNA was performed using the Trizol reagent (Thermo Fisher Scientific) and it was processed for qPCR analyses of *CXCL8* expression as described previously^21^. Relative gene expression was calculated using the 2^∆Ct^ method with ACTB as reference gene.

### Activity of the solubilized MMP-9

MMP enzymatic activities were determined by zymography using a 10% SDS-PAGE gel with 1% gelatin under non-denaturing conditions. After that, the gel was incubated with developing buffer and dyed with Coomassie as detailed by Cano-Garrido *et al*.^18^. Densitometry analyses of the bands were performed with the Image J software. The standard (kindly provided by the Laboratory of Immunobiology of the Rega Institute for Medical Research, KU Leuven, Belgium) corresponds to a mixture of purified monomeric MMP-9, multimeric MMP-9 and a mutant MMP-9 with a domain deletion^41^. The enzyme precursors, proMMP-9, are present in this standard.

### Statistical analysis

All data were analyzed using a mixed-effects model that accounted for the random effects of replicate (n = 3) and the fixed effects of treatment and/or time of sampling (JMP, SAS Institute Inc.). Sampling time entered the model as a repeated measure using an autoregressive covariance matrix. When more than 2 means were compared, differences were established using the Tukey’s multiple mean separation test. Data were previously transformed to achieve a normal distribution when necessary. Results are expressed as the means of non-transformed data ± standard error of the mean (SEM).

## Electronic supplementary material

Supplementary Material

## Data Availability

The datasets generated during and/or analysed during the current study are available from the corresponding author on reasonable request.
