# Supplementary material for: A new approach to obtain pure and active proteins from Lactococcus lactis protein aggregates
Source: Sci Rep. 2018 Sep 17;8:13917. doi: 10.1038/s41598-018-32213-8 (PMC6141594; doi:10.1038/s41598-018-32213-8)

A new approach to obtain pure and active proteins from *Lactococcus lactis* protein aggregates

L Gifre-Renom, O Cano-Garrido, F Fàbregas, R Roca-Pinilla, J Seras-Franzoso, N Ferrer-Miralles, A Villaverde, À Bach, M Devant, A Arís and E Garcia-Fruitós

SUPPLEMENTARY MATERIAL

Supplementary Figure 1. Original western blots (top) and Coomassie stained gels (bottom) for the M-SAA3 purification conditions stated in Table 1. In the left, the respective cropped lanes shown in Figure 2 where high-contrast was applied when necessary -only in Coomassie lanes- to allow a better display of the bands. Numbered lanes correspond to eluted fractions along the imidazole gradient. *D* dialyzed samples; *EP* eluted protein; *FT* flow through; *M* protein marker.

A. Condition 1

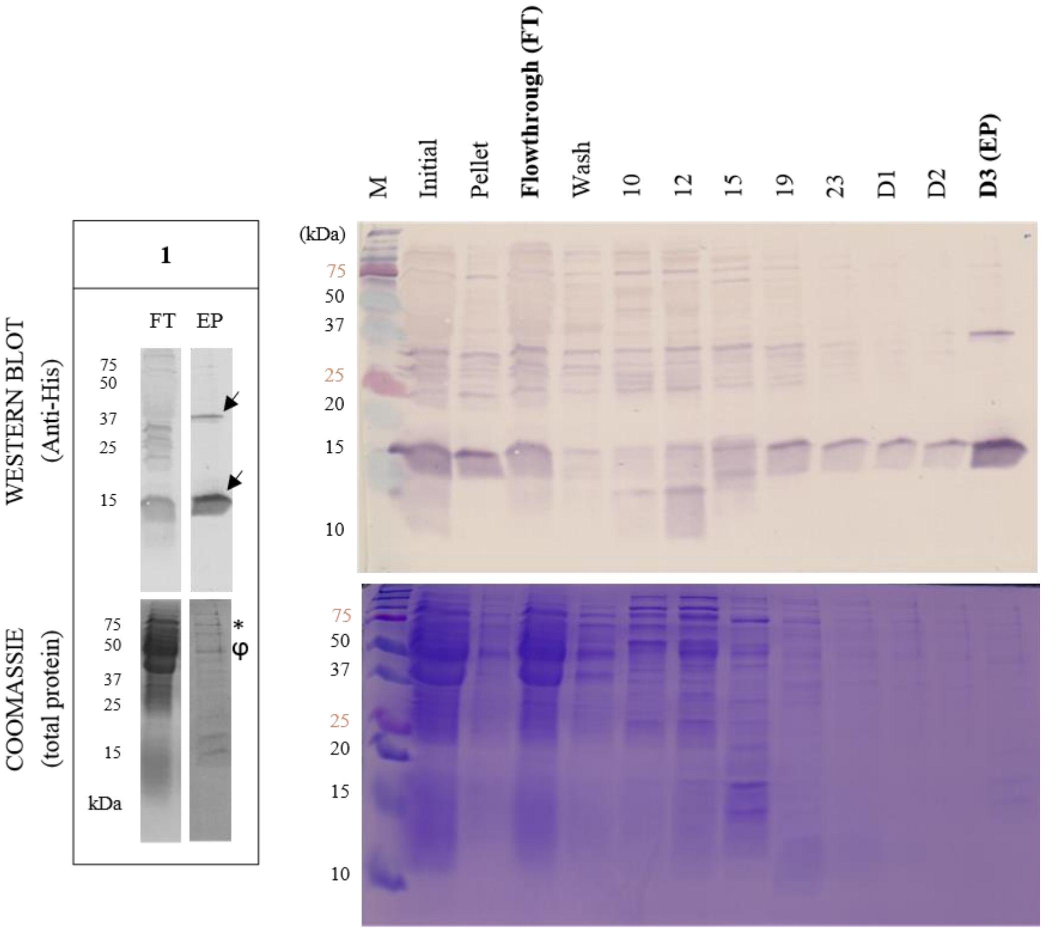

B. Condition 2

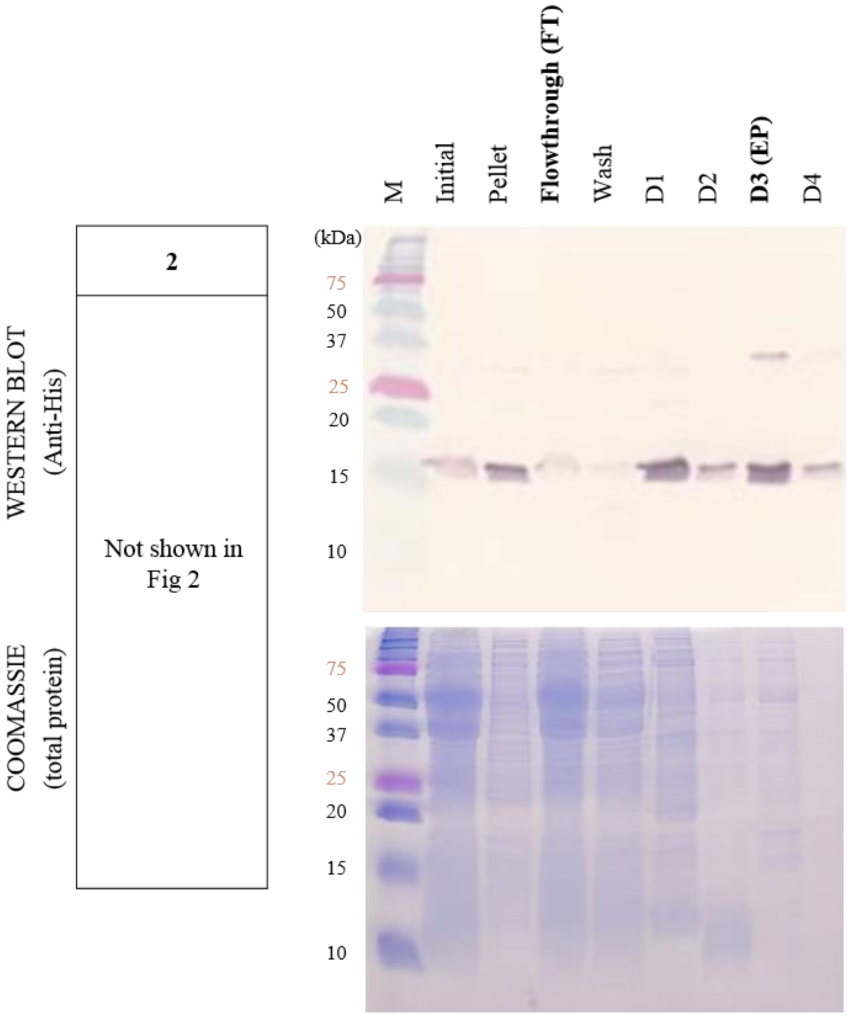

C. Condition 3

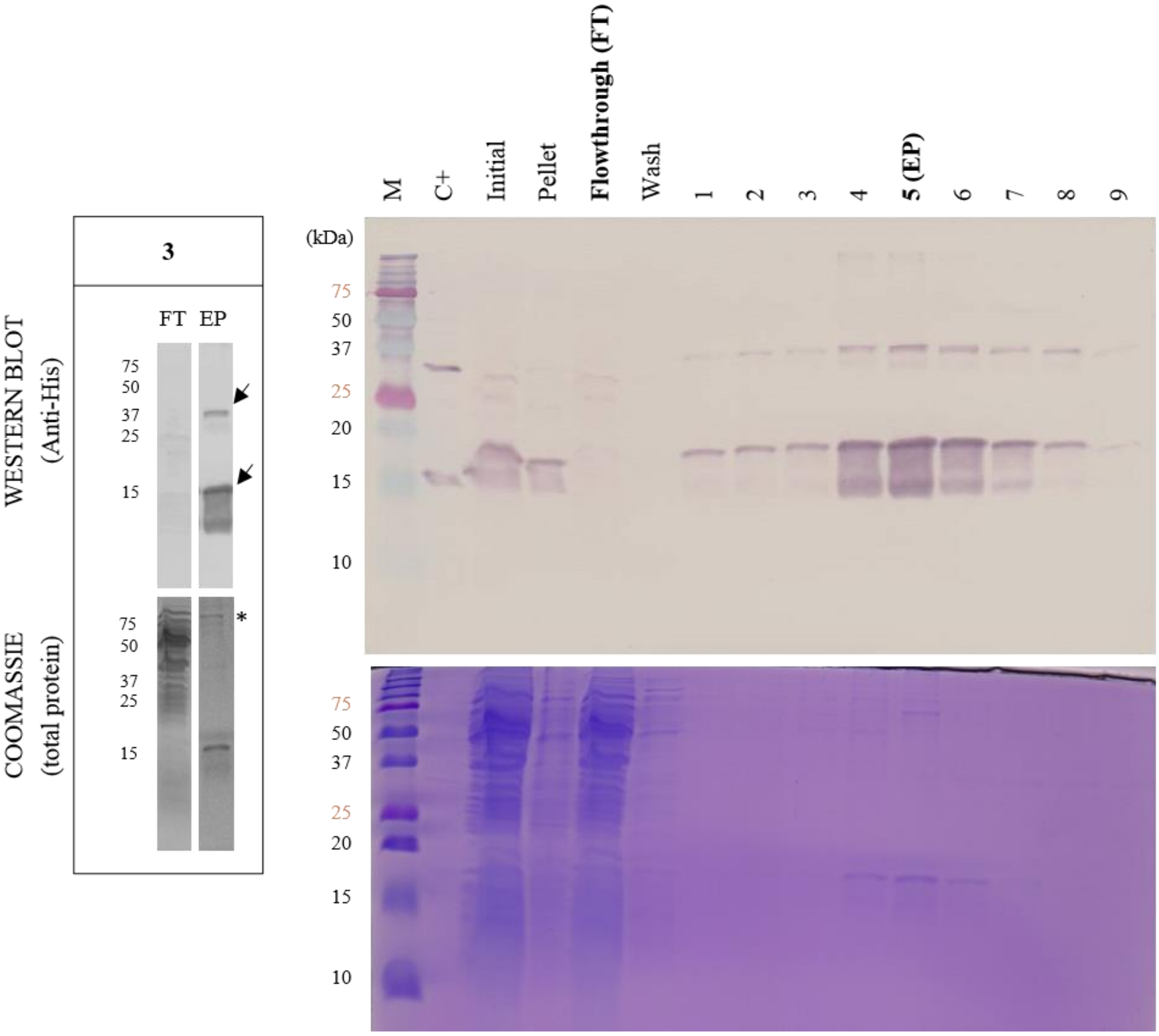

D. Condition 4

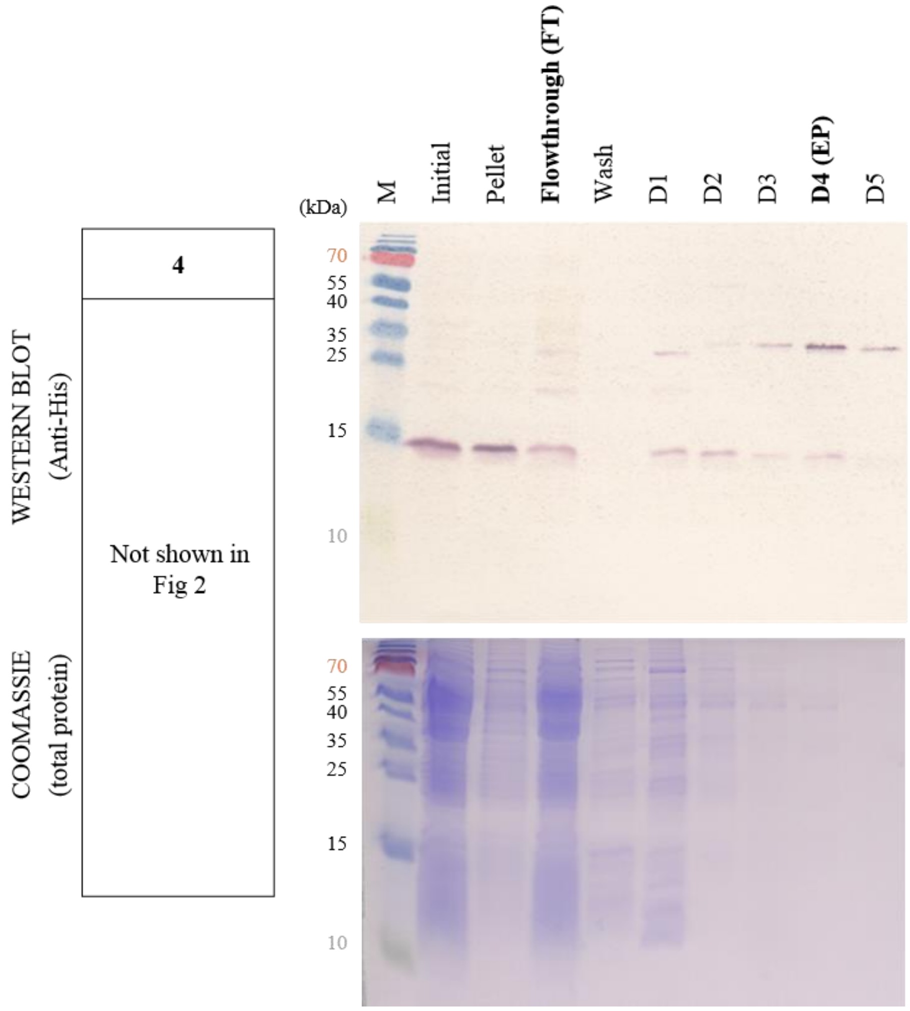

E. Condition 5

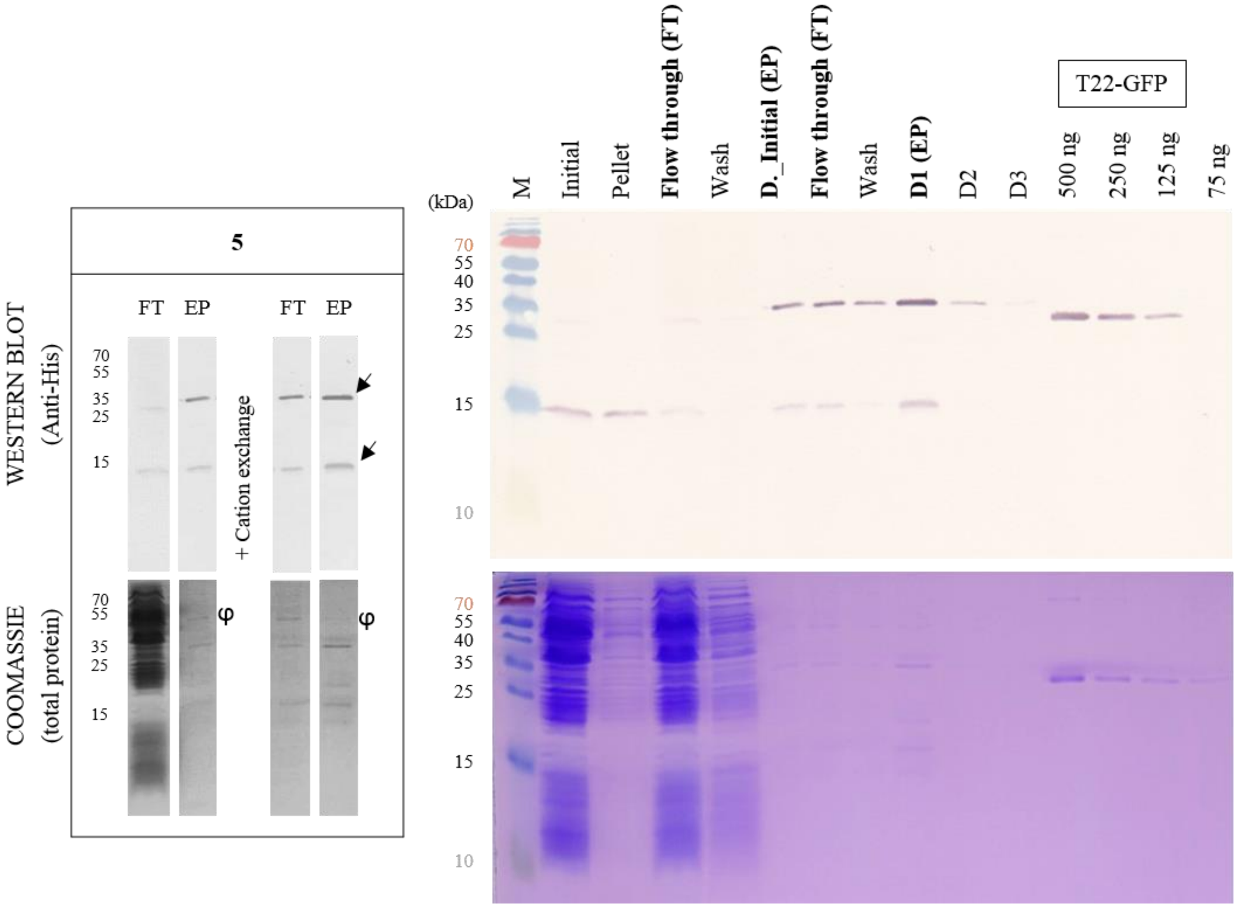

F. Condition 6

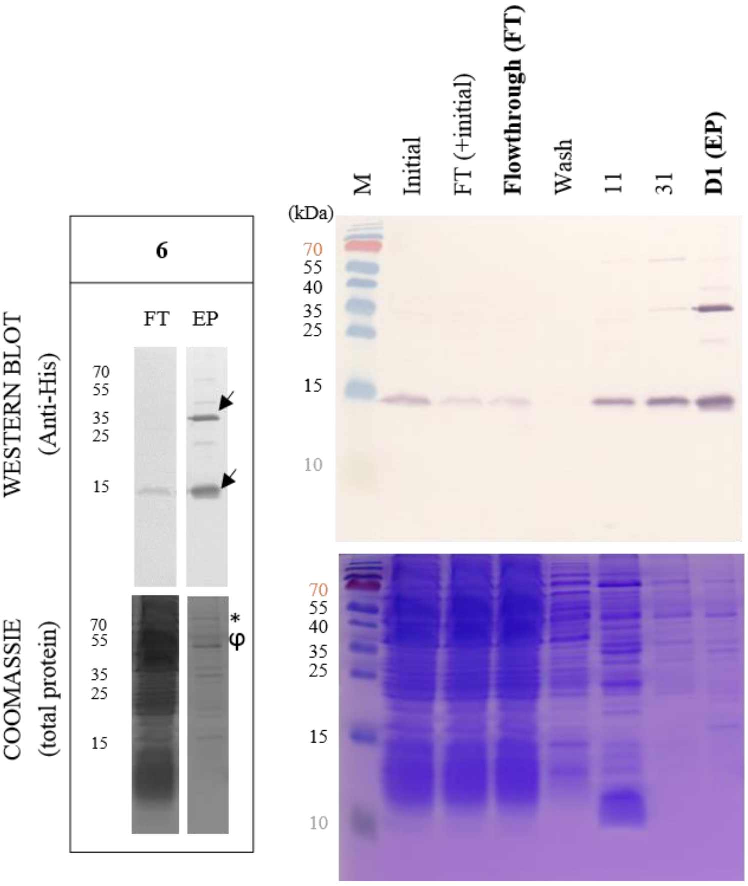

G. Condition 7

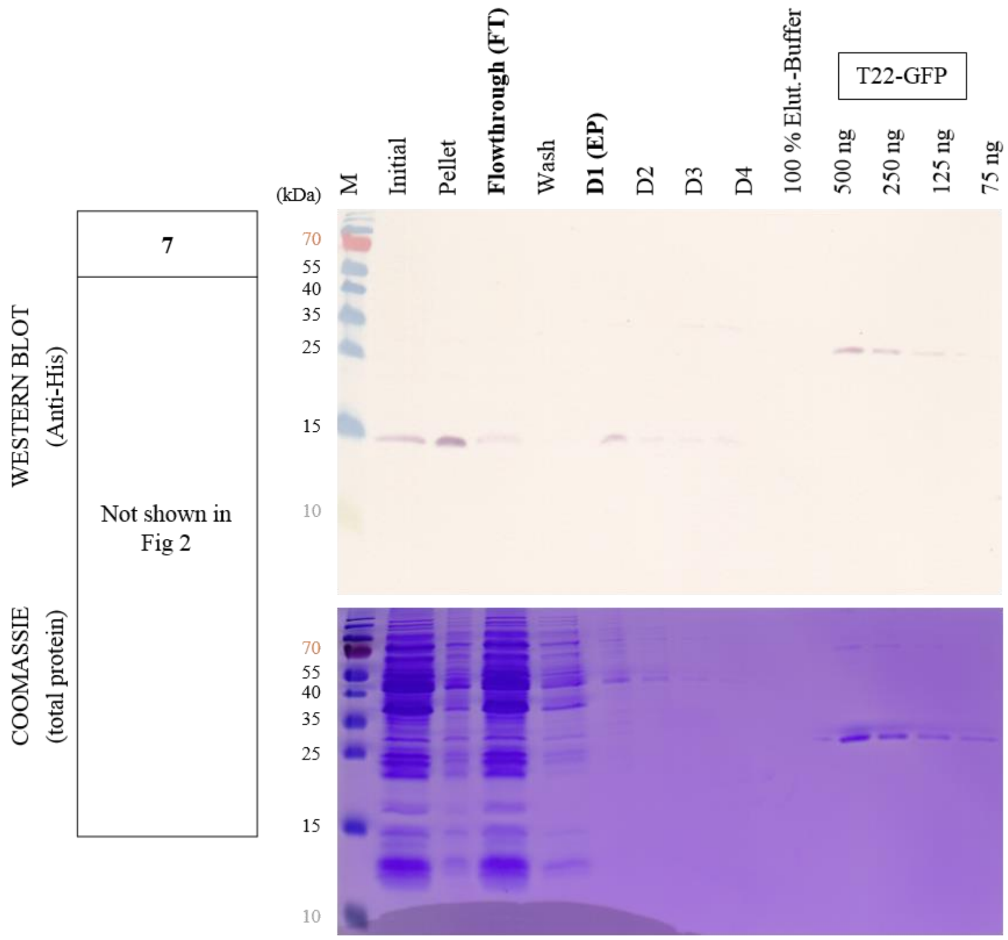

H. Condition 8

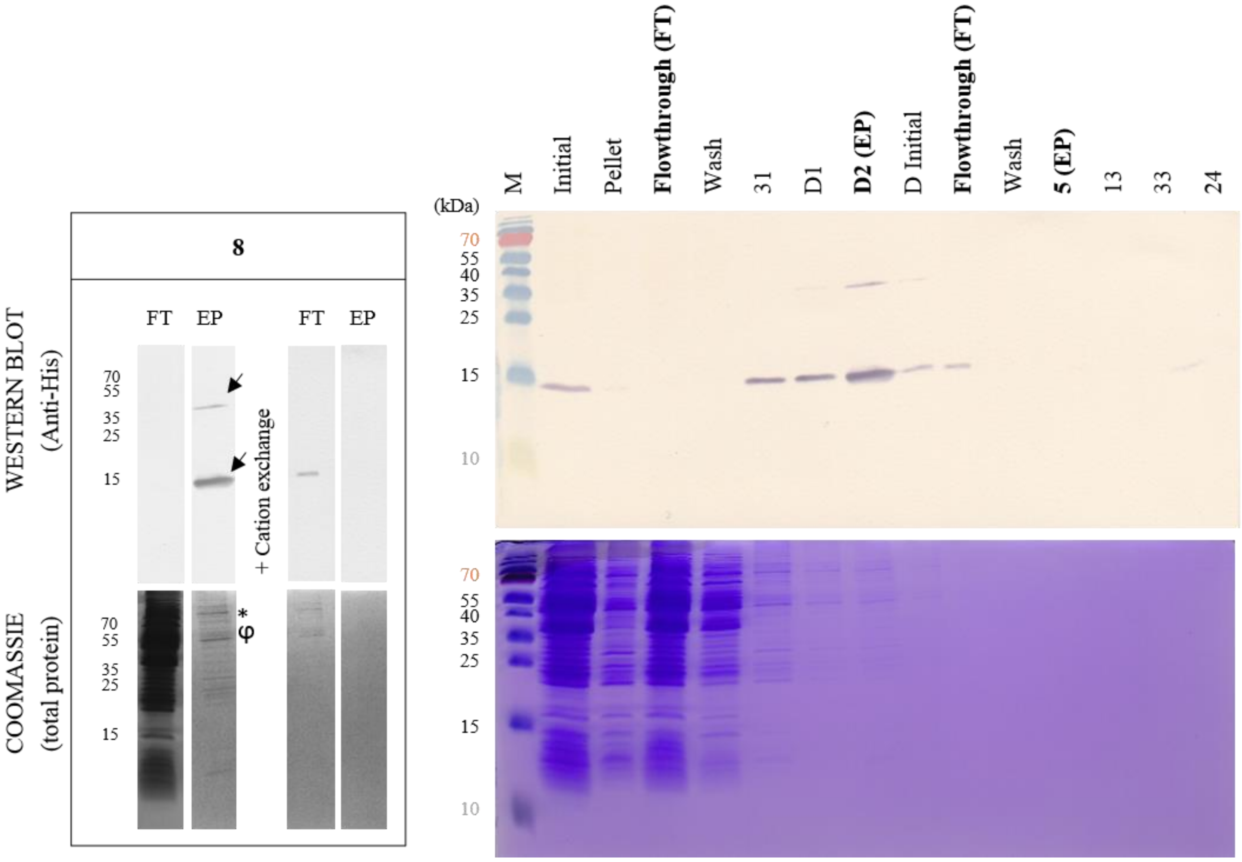

Supplementary Figure 2. Original western blots (top) and Coomassie stained gels (bottom) for the purification of the solubilized M-SAA3 and MMP-9. In the left, the respective cropped lanes shown in Figure 4. *CMS* Coomassie stained gel; *EP* eluted protein; *FT* flow through; *M* protein marker; *SN* supernatant; *WB* western blot.

A. Solubilized M-SAA3

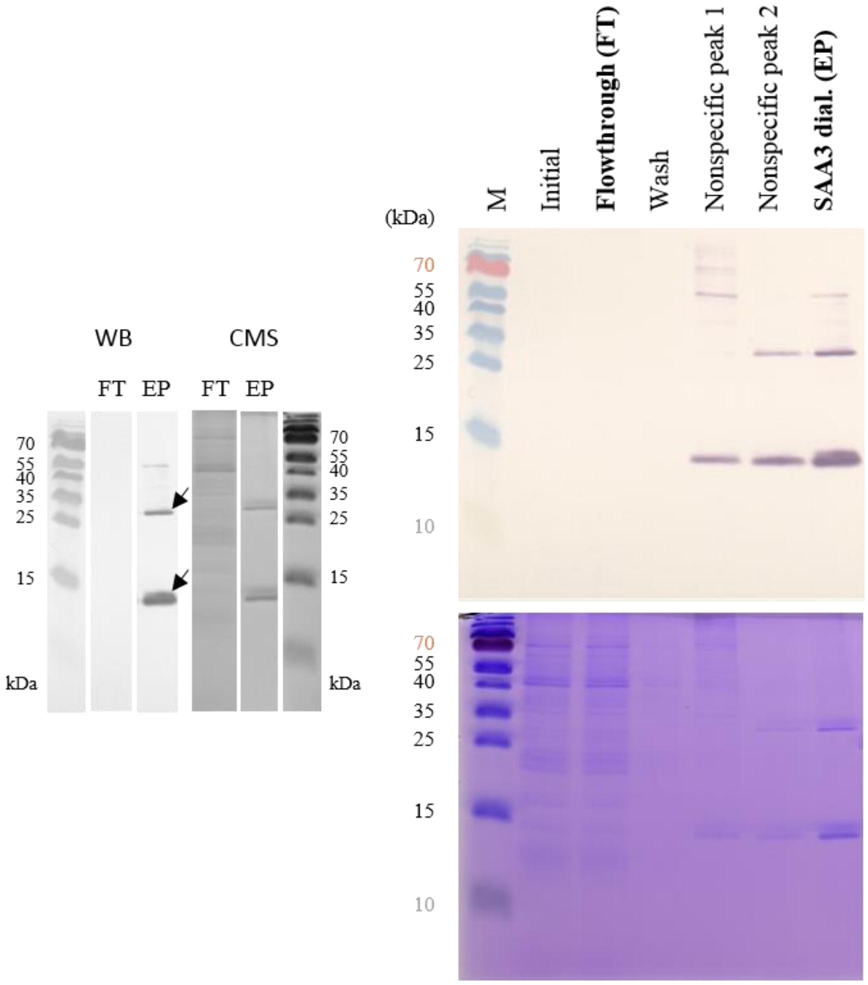

B. Solubilized MMP-9

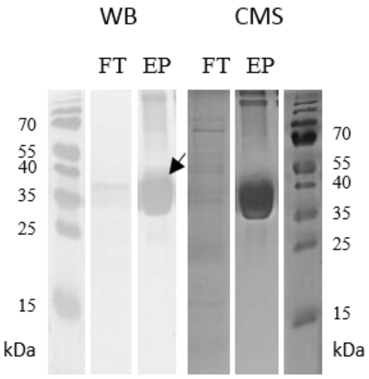

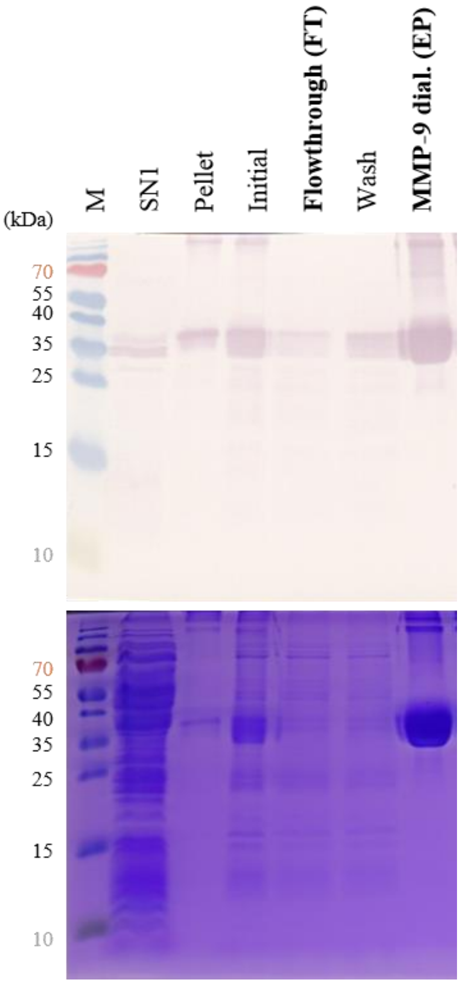

Supplement: Supplementary file 1 — Supplementary Material [file 41598_2018_32213_MOESM1_ESM.pdf]
